# Signalling pathways activated by glucagon-like peptide-1 (7-36) amide in the rat heart and their role in protection against ischaemia

**Published:** 2008-04

**Authors:** Barbara Huisamen, Amanda Lochner, Sonia Genade

**Affiliations:** MRC Cape Heart Centre and Department of Biomedical Sciences, Division of Physiology, Faculty of Health Sciences, University of Stellenbosch, Tygerberg; MRC Cape Heart Centre and Department of Biomedical Sciences, Division of Physiology, Faculty of Health Sciences, University of Stellenbosch, Tygerberg; Department of Biomedical Sciences, Division of Physiology, Faculty of Health Sciences, University of Stellenbosch, Tygerberg.

## Abstract

**Summary:**

Glucagon-like peptide-1 is an incretin hormone proposed to have insulinomimetic effects on peripheral insulin-sensitive tissue. We examined these effects on the heart by using isolated, perfused rat hearts and adult ventricular myocytes. During normoxic perfusion, no effects of escalating concentrations of GLP-1 on either heart rate or left ventricular developed pressure were found. With functional performance as readout, we found that GLP-1 directly protected the heart against damage incurred by global low-flow ischaemia. This protection was sensitive to the presence of iodo-acetate, implicating activation of glycolysis, and was abolished by wortmannin, indicative of PI-3-kinase as mediator of protection. In addition, GLP-1 had an infarct-sparing effect when supported by the presence of the dipeptidyl peptidase-IV inhibitor valine pyrrolidide.

GLP-1 could not directly activate protein kinase B (also called Akt) or the extracellular regulated kinases Erk1/2 in hearts or cardiocytes under normoxic conditions, but phosphorylation of the AMP-activated kinase (AMPK) on Thr^172^ was enhanced. In addition, the glycolytic enzyme phosphofructokinase-2 was activated dose dependently. During reperfusion after ischaemia, modulation of the phosphorylation of PKB/Akt as well as AMPK was evident. GLP-1 therefore directly protected the heart against low-flow ischaemia by enhancing glycolysis, probably via activation of AMP kinase and by modulating the profile of activation of the survival kinase PKB/Akt.

## Summary

Glucagon-like peptide-1 (GLP-1) is an incretin hormone secreted rapidly from the intestine in response to nutrient ingestion.^1^ The plasma levels of this hormone are tightly regulated by the enzyme dipeptidyl peptidase IV.^2^

GLP-1 plays a role in regulating plasma glucose levels by enhancing insulin secretion from the pancreatic beta-cells^3^ in conjunction with diminishing levels of glucagon and somatostatin. Insulin release is mediated via activation of a specific GLP-1 receptor that is linked to activation of adenylyl cyclase and a subsequent rise in cAMP and activation of PKA. These signalling events effect a rise in intracellular Ca^2+^ as trigger for insulin secretion through closure of the K_ATP_ channels. GLP-1 has turned out to be the most potent and efficient regulatory stimulator of insulin secretion discovered thus far. Clinical studies investigating type 2 diabetes revealed that GLP-1 is strongly insulinotrophic, even in patients with long-standing illness and secondary sulphonylurea failure.^4^

Recent evidence suggests also that GLP-1 has peripheral effects to enhance glucose utilisation in insulin-sensitive tissues (ie, fat, muscle and liver).^5^,^6^ It stimulates glycogen synthesis and increases glycolysis and glucose oxidation in the liver,^7^ isolated rat soleus muscle^8^ and adipocytes,^9^ as well as in human skeletal muscle.^10^ Specific receptors for GLP-1 have been described in adipocytes,^11^ lung tissue,^12^ liver^13^ and gastric mucosa^14^ while northern blot analysis revealed the presence of mRNA for the receptor protein in diverse tissues, including the heart.^15^,^16^ Cloning and sequencing of the heart isoform furthermore revealed the same deduced amino acid sequence as the pancreatic receptor.^16^ However, analysis of the provoked signalling of GLP-1 binding to peripheral tissue^17^,^18^ or cells transfected with receptor protein^17^ argues for a different response than the cAMP−PKA pathway evoked in beta-cells, pointing to the possible activation of multiple signalling pathways.

GLP-1 infusion in rats resulted in myocardial pressor effects^19^,^20^ while infusion in humans after acute myocardial infarction afforded improved left ventricular function.^21^ Therapy for type 2 diabetics based on the actions of GLP-1 is hailed as one of the most promising recent approaches to this disease. In view of the vulnerability of the heart in type 2 diabetes and the documented effects of GLP-1 on pancreatic as well as peripheral tissue, we aimed to investigate the myocardial effects of GLP-1 using isolated, perfused rat hearts and adult ventricular myocytes.

## Materials and methods

Male Wistar rats (225−250 g) were used in this study. The animals had free access to food and water and were kept on a 12-hour day/night cycle in an AAALAC-accredited facility. Rats were anaesthetised by intraperitoneal injection of sodium pentobarbital (160 mg/kg). The study conformed to the *Guide for the Care and Use of Laboratory Animals* of the NIH (Publication No. 85-23, revised 1996).

GLP-1 (7-36) amide (Sigma) was used in this study and is henceforth indicated as GLP-1. Wortmannin, glucose-6-phosphate, fructose-6-phosphate, pyrophosphate-sensitive PFK and pyrophosphate reagent were from Sigma, collagenase type 2 was from Worthington, bovine serum albumin (BSA) (fraction V, fatty acid free) from Boehringer Mannheim and all antibodies from Cell Signaling. The DPP-IV inhibitor, valine pyrrolidide, was kindly supplied by Novo Nordisk, Denmark. All other chemicals were of the highest grade commercially available.

## Isolated heart perfusions

After anaesthesia, the hearts were quickly excised and arrested in ice-cold Krebs–Henseleit solution (KR) and perfused retrogradely. 22 They were fitted with an intraventricular balloon to monitor left ventricular pressure.

**Fig. 1.**
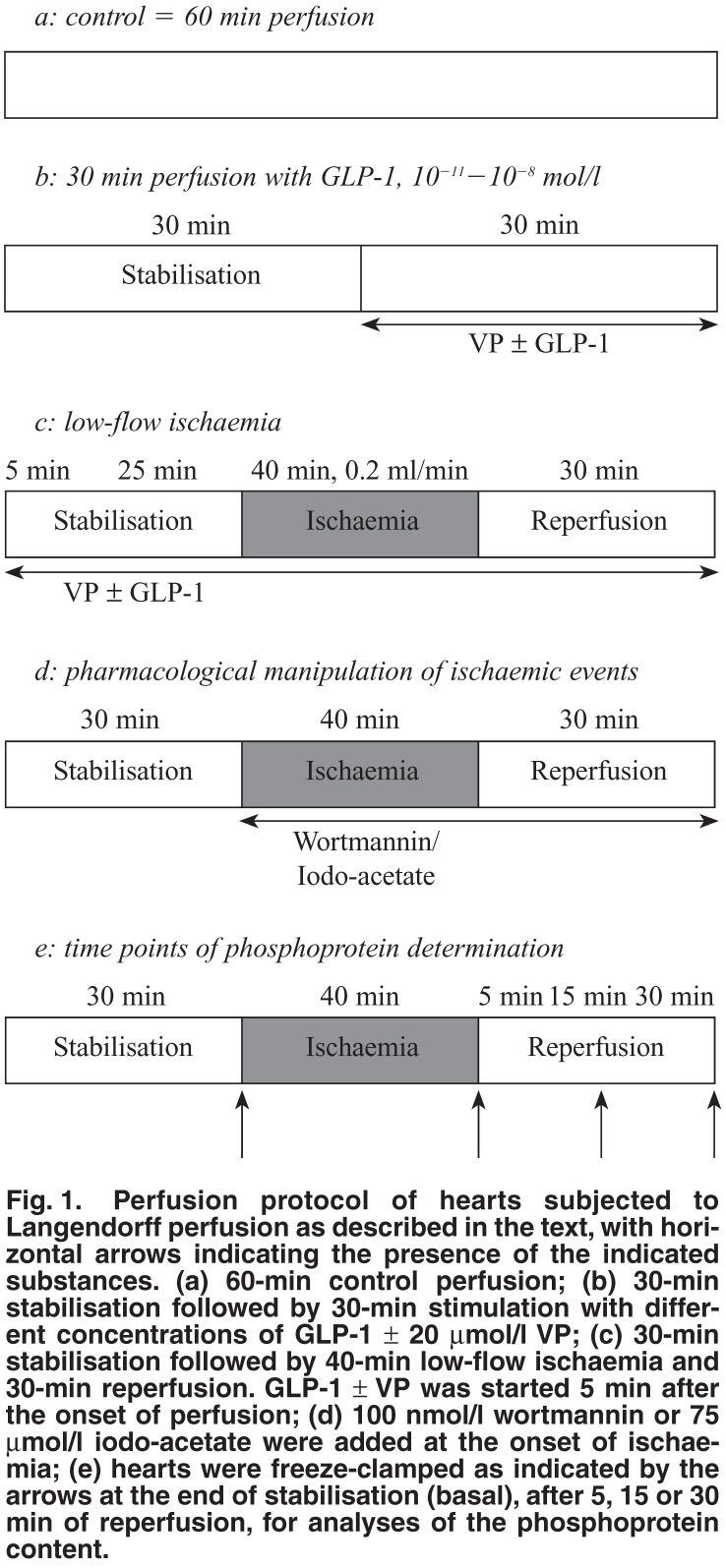
Perfusion protocol of hearts subjected to Langendorff perfusion as described in the text, with horizontal arrows indicating the presence of the indicated substances. (a) 60-min control perfusion; (b) 30-min stabilisation followed by 30-min stimulation with different concentrations of GLP-1 ± 20 μmol/l VP; (c) 30-min stabilisation followed by 40-min low-flow ischaemia and 30-min reperfusion. GLP-1 ± VP was started 5 min after the onset of perfusion; (d) 100 nmol/l wortmannin or 75 μmol/l iodo-acetate were added at the onset of ischaemia; (e) hearts were freeze-clamped as indicated by the arrows at the end of stabilisation (basal), after 5, 15 or 30 min of reperfusion, for analyses of the phosphoprotein content.

The myocardial temperature and left ventricular developed pressure (LVDevP) were monitored throughout the experiment as reported previously.^22^ Global low-flow ischaemia was induced by reducing the coronary flow rate from 11.4 ± 0.4 ml/min to 0.2 ml/min, by means of a Gilson Minipuls 2 peristaltic pump. The hearts were perfused according to the protocol indicated and described in Fig. 1, freeze-clamped at the times indicated and stored in liquid nitrogen for biochemical analyses.

## Infarct size determination

The infarct size after regional ischaemia was determined after coronary artery ligation for 35 min, followed by reperfusion for 30 min as described previously,^22^,^23^ and expressed as a percentage of the area at risk.

## Preparation of myocytes

Calcium-resistant adult ventricular myocytes in an unstimulated state were prepared as described previously.^24^ After isolation, the myocytes were suspended in a buffer containing, in mmol/l: HEPES, 10; KCl, 6; NaH_2_PO_4_, 0.2; Na_2_HPO_4_, 1; MgSO_4_, 1.4; NaCl, 128; pyruvate, 2; glucose, 5.5; 2% BSA (fraction V, fatty acid free) plus 1.25 mmol/l calcium, pH 7.4. The cells were left for one to two hours under an oxygen atmosphere on a gently shaking platform to recover from the trauma of isolation. This procedure routinely rendered in excess of 80% viable cells as measured by trypan blue exclusion.

After recovery, the cells were spun down into a loose pellet (3 min at 100 rpm), and washed twice with and suspended in a suitable volume of the above medium from which pyruvate was omitted (buffer A).

## Cyclic AMP determination

For tissue cyclic AMP analysis, 100−200 mg of frozen tissue was extracted with 1.2 ml 6% perchloric acid. Extracts were neutralised and cAMP content was determined using a commercial kit (Amersham) according to the manufacturer’s instructions. A standard curve ranging from 0.125−16 pmol cAMP was included in each assay.

## Immunoblotting for determination of phosphorylated kinases

Frozen tissue was lysed (polytron PT-10 homogeniser; 2 × 4 sec, setting 4) and myocytes were sonicated on ice in buffer containing, in mmol/l: tris-HCl, 20 (pH 7.5); EGTA, 1; EDTA, 1; NaCl, 150; Na_2_VO_3_, 1; beta-glycerophosphate, 1; sodium-pyrophosphate, 2.5; PMSF, 0.3; Triton X-100 1% (v/v) plus 10 μg/ml leupeptin and aprotinin, respectively. Lysates were cleared from particulate matter by spinning for 15 min at 14 000 rpm in a microfuge. Equal amounts of cytosolic proteins^25^ were separated on a 10% SDS poly-acrylamide gel and electro-transferred to Immobilon^TM^-P membranes.

The membranes were blocked for two hours in tris-buffered saline (TBS) containing 0.1% Tween-20 and 5% non-fat milk powder, after which they were probed for 16 hours with a primary antibody (Ser^473^- or Thr^308^-phosphorylated PKB/Akt, total PKB/Akt, GSKα/β as downstream substrate of PKB/Akt, Thr^172^-phosphorylated AMP-activated protein kinase or Erk1/2). Quantification was done by laser scanning densitometry and suitable software (UN-SCAN-IT, Silkscience). Care was taken to include a reference standard on all blots for comparison purposes.

## Determination of fructose-2,6-bisphosphate levels

Cardiomyocytes in buffer A were stimulated with different concentrations of GLP-1 by incubation under an oxygen atmosphere in a shaking water bath at 37°C for 30 min, where after the cells were loosely spun down, the supernatant was aspirated and the pellet washed twice with buffer A. Cells were lysed by sonicating on ice in the following buffer, in mmol/l: HEPES, 20 (pH 7.5); NaF, 20; KCl, 30; EDTA, 5; EGTA, 5; Na_2_VO_3_, 1; DTT, 1; benzamidine, 1; leupeptin, 4 μg/ml; and aprotinin, 1 μg/ml. The protein concentrations were determined,^25^ and lysate containing equal amounts of protein were mixed with an equal volume of 0.1 N NaOH and incubated for 5 min at 80°C.^26^ At this point, samples were stored at −80°C until assayed.

The lysates were then microfuged (10 min at 14 000 rpm at 4°C) and neutralised with 0.1 N CH_3_COOH. After neutralisation, the samples were centrifuged as above. Twenty-five microlitres of the supernatant was assayed for fructose-2,6-bisphosphate (fru-2,6-P_2_) content using its ability to stimulate PFK-1 in a coupled enzyme system containing 0.5 mmol/l pyrophosphate, pyrophosphate-sensitive fructose-6-phosphate kinase from potato tubers and a commercial pyrophosphate reagent cocktail. In this reaction, β-NADH is oxidised to NAD, which can be followed spectrophotometrically at A_340_ nm. The reaction was performed in a Beckman DU640 temperature-controlled spectrophotometer coupled to a PC with suitable software. Readings were taken at 20-sec intervals over a 3-min period and the rate of formation calculated as ΔA_340_ nm/20 sec.

## Statistical analyses

Data are presented as mean ± SEM. Comparisons between multiple groups were performed by one-way ANOVA, twoway ANOVA or repeated-measures ANOVA, followed by the Bonferroni *post-hoc* test (Graph-Pad Prism). A value of *p* < 0.05 was considered statistically significant.

## Results

Over a 30-min period, escalating concentrations of GLP-1 (10^-11^–10^-8^ mol/l) in the presence of 20 μmol/l valine pyrrolidide (VP) had no effect on either heart rate or left ventricular developed pressure (results not shown). In addition, there was no evidence of accumulation of cAMP in the tissue under these conditions (360.7 ± 11.1 control vs 373 ± 20.5 pmol/g ww).

**Fig. 2.**
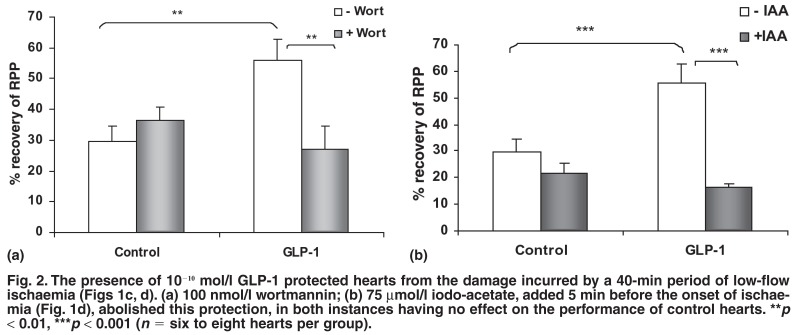
The presence of 10^-10^ mol/l GLP-1 protected hearts from the damage incurred by a 40-min period of low-flow ischaemia (Figs 1c, d). (a) 100 nmol/l wortmannin; (b) 75 μmol/l iodo-acetate, added 5 min before the onset of ischaemia (Fig. 1d), abolished this protection, in both instances having no effect on the performance of control hearts. **p < 0.01, ***p < 0.001 (*n* = six to eight hearts per group).

Isolated hearts were subjected to 40 min of normothermic low-flow (0.2 ml/min) ischaemia in the presence or absence of 10^-10^ mol/l GLP-1 and 20 μmol/l VP (Fig. 1c). The presence of GLP-1 resulted in a significantly improved rate pressure product (RPP) recovery on reperfusion (55.64 ± 6.83% of pre-ischaemic values vs 29.75 ± 4.97% without GLP-1) (Fig. 2a). Addition of the PI-3-kinase inhibitor wortmannin (100 nmol/l) before the onset of ischaemia abolished this protective effect (Fig. 2a). Similarly, the presence of 75 μmol/l iodo-acetate during the ischaemic period abolished protection while having no effect GLP-1 (Fig. 2b).

**Fig. 3.**
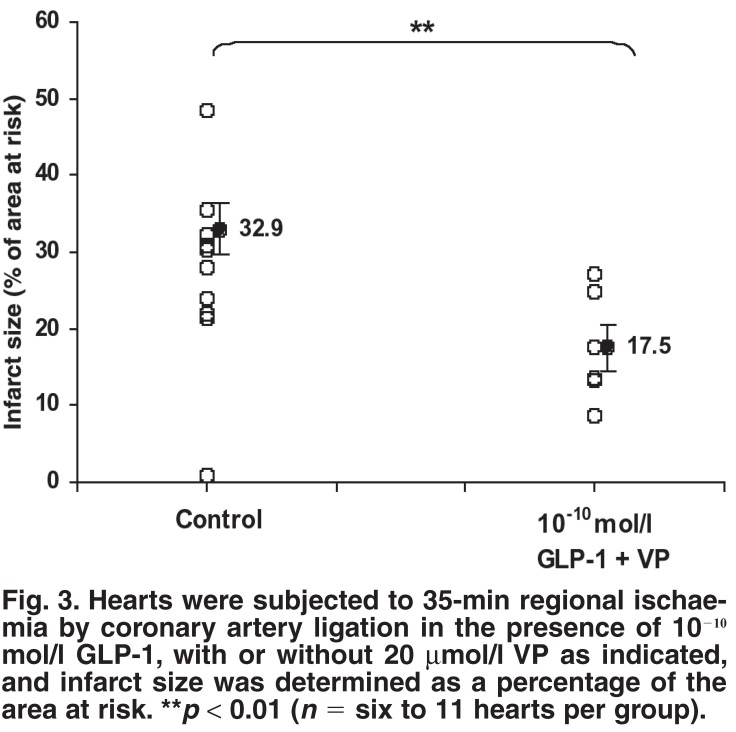
Hearts were subjected to 35-min regional ischaemia by coronary artery ligation in the presence of 10^-10^ mol/l GLP-1, with or without 20 μmol/l VP as indicated, and infarct size was determined as a percentage of the area at risk. **p < 0.01 (*n* = six to 11 hearts per group).

As shown in Fig. 3, 10^-10^ mmol/l GLP-1, in the presence of the DPP-IV inhibitor VP (20 μmol/l), significantly reduced infarct size in comparison to control hearts, when expressed as a percentage of the area at risk. The presence of VP did not alter the infarct size of control hearts. The area at risk was similar in control vs treated hearts (56.02 ± 2.25 vs 54.13 ± 2.21%, *n* = 6).

## Signalling pathways involved in GLP-1 actions

**Fig. 4.**
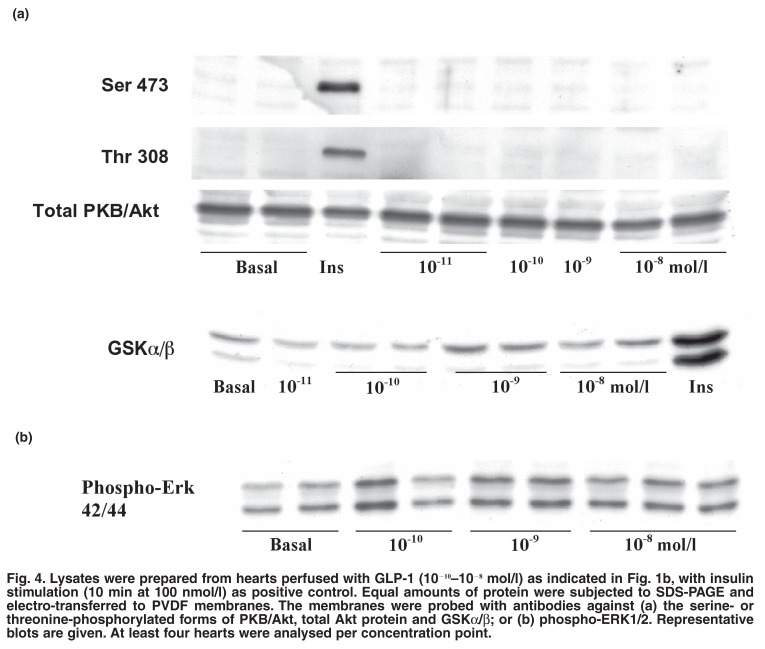
Lysates were prepared from hearts perfused with GLP-1 (10^-10^–10^-8^ mol/l) as indicated in Fig. 1b, with insulin stimulation (10 min at 100 nmol/l) as positive control. Equal amounts of protein were subjected to SDS-PAGE and electro-transferred to PVDF membranes. The membranes were probed with antibodies against (a) the serine- or threonine-phosphorylated forms of PKB/Akt, total Akt protein and GSKα/β; or (b) phospho-ERK1/2. Representative blots are given. At least four hearts were analysed per concentration point.

The results presented in Fig. 2a show that the protection afforded by GLP-1 against ischaemic damage involved the well-known pro-survival kinase PI-3-kinase. However, in hearts perfused for 30 min under normoxic conditions with GLP-1 (10^-11^–10^-8^ mol/l) (Fig. 1e), no evidence of phosphorylation of PKB/Akt or GSKα/β as downstream substrates of this pathway could be found (Fig. 4a). Similarly, in the cardiomyocytes, there was no evidence of phosphorylation of these proteins after stimulation with the same concentrations of GLP-1 (results not shown). The extracellular regulated kinase Erk1/2 was also not activated in hearts or myocytes stimulated under these conditions (Fig. 4b).

**Fig. 5.**
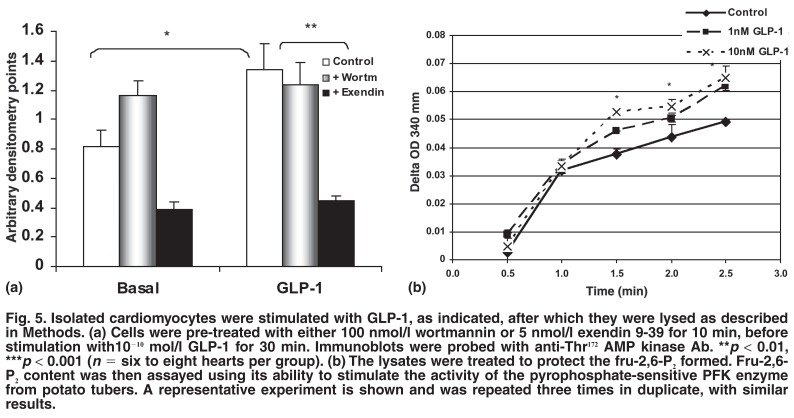
Isolated cardiomyocytes were stimulated with GLP-1, as indicated, after which they were lysed as described in Methods. (a) Cells were pre-treated with either 100 nmol/l wortmannin or 5 nmol/l exendin 9-39 for 10 min, before stimulation with10^-10^ mol/l GLP-1 for 30 min. Immunoblots were probed with anti-Thr^172^ AMP kinase Ab. **p < 0.01, ***p < 0.001 (*n* = six to eight hearts per group). (b) The lysates were treated to protect the fru-2,6-P_2_ formed. Fru-2,6- P_2_ content was then assayed using its ability to stimulate the activity of the pyrophosphate-sensitive PFK enzyme from potato tubers. A representative experiment is shown and was repeated three times in duplicate, with similar results.

**Fig. 6.**
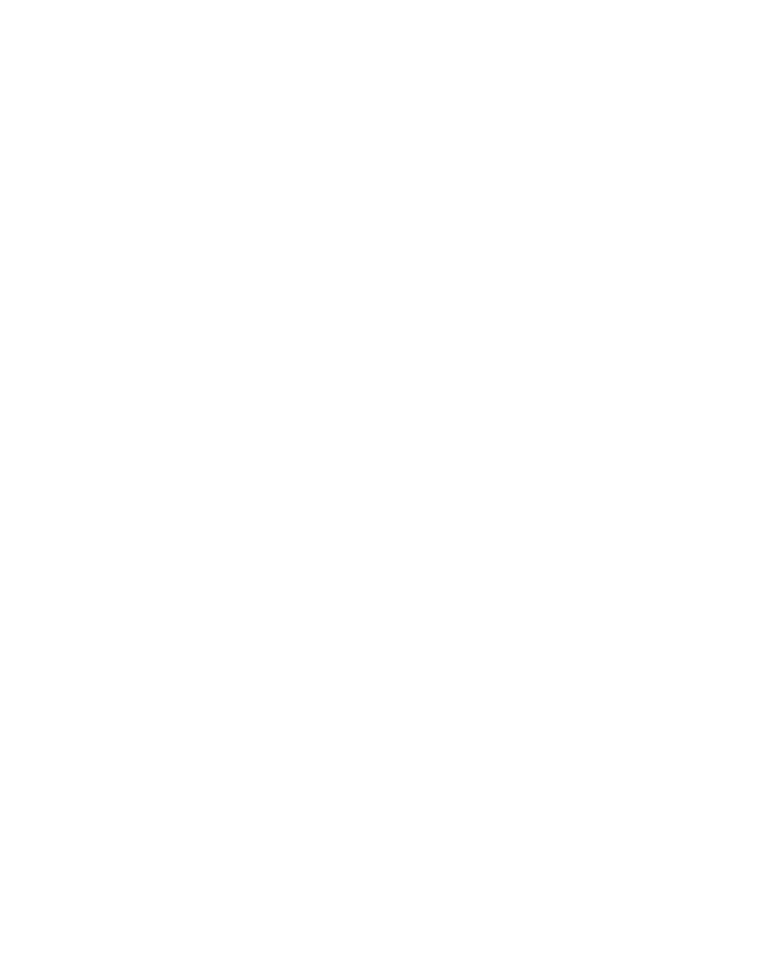
Hearts were perfused with or without GLP-1, as indicated in Fig. 1c and freeze-clamped at the indicated time points (Fig. 1e) for analysis of the phosphoprotein content by western blotting, as described in Methods. Phosphorylation of (a) Ser^473^ on PKB/Akt; and (b) Thr172 on AMPK were measured. *p < 0.05, **p < 0.01 and ***p < 0.001 vs basal levels within group. ^#^*p* < 0.05 vs control. Four to eight individual hearts were analysed per time point.

Under normoxic perfusion conditions, therefore before the onset of ischaemia, as low as 10^-11^ mmol/l GLP-1 resulted in a significant phosphorylation of AMP-activated kinase on Thr^172^ (Figs 5a, 6b). In cardiomyocytes, this effect could be abolished by the presence of 5 nmol/l exendin 9-39, a specific antagonist of GLP-1, but not by wortmannin (Fig. 5a). Furthermore, as shown in Fig. 5b, dose-dependent activation of the enzyme phosphofructokinase-2 occurred, as measured by accumulation of fru-2,6-P_2_ and the ability of the latter to stimulate the activity of PFK-1.

To understand the involvement of the PI-3K, PKB/Akt or the AMPK signalling pathways in the protective effects of GLP-1 against ischaemia, we documented the profile of the phosphorylation state of these kinases during low-flow ischaemia and the reperfusion phases. Contrary to expectation, the presence of GLP-1 had no effect on the activation of PKB/Akt during the ischaemic phase and attenuated its activation during reperfusion (Fig. 6a). Although activation of PKB/Akt was always lower in the presence of GLP-1 during reperfusion, the temporal pattern of activation was similar to the hearts perfused in the absence of GLP-1, declining during ischaemia, with a significant peak of activation after 15 min reperfusion, and again declining towards 30 min reperfusion.

AMPK, as expected, showed strong activation during ischaemia that increased with time. Again the presence of GLP-1 did not alter this. However, during reperfusion, in contrast to the lower PKB/Akt activation compared to control hearts, observed in the presence of 10^-10^ mol/l GLP-1, Thr^172^ phosphorylation of AMPK was consistently enhanced above control values. We furthermore observed a peak of activation of this kinase at 15 min reperfusion (Fig. 6b).

## Discussion

GLP-1 or substances such as the DPP-IV inhibitor valine pyrrolidide, which probably act via GLP-1,^27^ are currently hot topics of attention as possible antidiabetic agents. Some of these substances have already passed phase 3 clinical trials and are used in practice. Recently published data show that GLP-1 promotes peripheral glucose utilisation^5^,^6^ but, more importantly, Nikolaidis *et al.*^21^ reported that infusion of GLP-1 as added therapy gave additional benefit to patients with acute myocardial infarction and severe systolic dysfunction after primary angioplasty. Because of the latter, this study set out to investigate the effects of GLP-1 on the isolated, perfused rat heart in conjunction with adult ventricular myocytes.

During normoxic perfusion conditions, we found no direct effect of GLP-1 (10^-11^–10^-8^ mol/l) in the presence of VP on either heart rate or left ventricular developed pressure (results not shown). This supports the conclusion of previous studies that myocardial pressor effects of GLP-1 are solely of autonomic origin.^19^,^20^ The DPP-IV inhibitor valine pyrrolidide did not alter any of the measured parameters and all control hearts were therefore perfused in the presence of 20 μmol/l VP.

Using the isolated, perfused heart, we demonstrated that the presence of 10^-10^ mol/l GLP-1, besides having an infarct-sparing effect, afforded protection against global low-flow ischaemia, as measured by the RPP as an index of functional recovery. This improved recovery was because of enhanced glycolysis, since the presence of iodo-acetate abolished the protection. Moreover, our results indicated that the protective effects were mediated by PI-3-kinase, as reflected by inhibition of the protection afforded by wortmannin. During low-flow ischaemia, a model recognised as more representative of what happens during infarction *in vivo*, oxidation of glycogen becomes an important energy source,^28^ underscoring the conclusion that this is a relevant part of the protective effects of GLP-1.

In spite of the above results, GLP-1 did not activate PKB/Akt as downstream substrate of PI-3-kinase, as evidenced by a lack of phosphorylation of this kinase on either Ser^473^ or Thr^308^ or of its substrate, GSK-3α/β, in myocytes or perfused hearts (Figs 4a, b). This corroborates findings presented by Zhao *et al*.,^29^ who also could not demonstrate phosphorylation of PKB/Akt after stimulation of hearts with GLP-1. According to our results, the presence of GLP-1 attenuated the phosphorylation of PKB/Akt normally seen on reperfusion of a heart after an ischaemic period (Fig. 6a). These results therefore implicate a different downstream effector of PI-3-K to be involved as mediator of protection.

AMP-activated kinase also stimulates glycolysis in the heart by direct activation of PFK-2.^30^ As shown in Fig. 6b, GLP-1 treatment resulted in enhanced phoshorylation of AMPK in normoxic, perfused hearts. In addition, in isolated cardiomyocytes, GLP-1 stimulated activation of PFK-2, as evidenced by a rise in fru-2,6-P_2_ levels (Fig. 5b). These results may therefore also explain the protection against ischaemic damage observed in hearts perfused with GLP-1.

It is well recognised that the effects of AMPK are not abolished by wortmannin. Underscoring this, wortmannin could not abolish AMPK phosphorylation after GLP-1 treatment of cardiomyocytes, whereas the specific inhibitor, exendin 9-39 could do so (Fig. 5a). In addition, exendin 9-39 attenuated basal levels of AMPK activation. This indicates that either the inhibitor has effects independent of GLP-1 signalling, or that GLP-1 tonically regulates AMPK activation in cardiomyocytes.

Opposite to the effects noted on PKB/Akt activation, AMPK was consistently in a higher phosphorylated state during the reperfusion phase after ischaemia in the presence of GLP-1, with strong activation noted at 15 min reperfusion. The effects of GLP-1 on PKB/Akt and AMPK phosphorylation may be a direct or indirect effect as cross talk between the two kinases has been described previously.^31^

Although we found no evidence of cAMP accumulation in hearts perfused with GLP-1 under normoxic conditions, and our results for p42/44 ERK activation are not conclusive for activation of this kinase, the work of Bose *et al.*^32^ demonstrated a role for both in the actions of GLP-1 to protect against ischaemia. Raised cAMP is a well-documented occurrence during myocardial ischaemia, while the above study showed that inhibition of activation of the p42/44 ERK abolished the protection afforded by GLP-1.^32^ Our results, in turn, argue that GLP-1 modulates the response of the heart to ischaemia/reperfusion with regard to the survival kinase profile activated, but does not directly activate these kinases. We could, however, inhibit ERK activation with the MEK inhibitor PD098059 in future, to clarify the role of ERK in the protection elicited by GLP-1.

## Conclusion

This study showed that GLP-1 activated AMPK in the hearts and could protect the heart against low-flow ischaemia by enhancing glycolysis, but that PKB/Akt was probably not involved in this protection. Furthermore, the extent of phosphorylation of both PKB/Akt and AMPK on reperfusion was altered by the presence of GLP-1. GLP-1 therefore altered the response of the heart to an ischaemic incident. To the best of our knowledge, this is the first study indicating a role for AMP kinase activation in the actions of GLP-1 on the heart.
